# Imaging of Urachal Adenocarcinoma: A Case Report

**DOI:** 10.1093/bjrcr/uaaf064

**Published:** 2025-12-05

**Authors:** V Pramod, S C Sanjay, R Dheepika

**Affiliations:** Department of Radiodiagnosis, Kempegowda Institute of Medical Sciences Hospital and Research Centre, Bangalore, Karnataka, 560002, India; Department of Radiodiagnosis, Kempegowda Institute of Medical Sciences Hospital and Research Centre, Bangalore, Karnataka, 560002, India; Department of Radiodiagnosis, Kempegowda Institute of Medical Sciences Hospital and Research Centre, Bangalore, Karnataka, 560002, India

**Keywords:** uracahal adenocarcinoma, bladder carcinoma, hematuria, urachus, urachal remnant, ultrasound, MRI, CECT

## Abstract

Adenocarcinoma of the urachus is a rare but highly aggressive malignancy that arises from the urachal remnant. Due to its non-specific symptoms and potential to mimic more common and benign conditions, accurate and early diagnosis through imaging is crucial. This report will discuss the role of imaging in urachal adenocarcinoma regarding the characteristics and diagnosis of this tumour, with a focus on CECT, MRI, and ultrasound for detection, staging, and treatment planning. Since urachal adenocarcinoma is a rare and complex disease, optimal results can be achieved only with a combined approach, where close cooperation among urologists, radiologists, oncologists, and pathologists is absolutely necessary. This investigation underlines the importance of increased awareness and expertise in the early and accurate imaging of this malignancy and advocates that optimal survival and quality of life can be best achieved in these patients by a well-coordinated, expert-driven approach.

## Introduction

Urachal adenocarcinoma represents a rare and highly aggressive cancer originating from the urachal remnant, a vestigial structure connecting the bladder to the umbilicus.^1^ During the fetal period, the urachus is an important embryological structure extending between the developing bladder and the allantois.^2^ Its development occurs between the 4th and 6th weeks of intrauterine life. Normally, it involutes by the 12th week of gestation and is usually present after birth in the form of a median umbilical ligament.^3^ However, remnants of the urachus may sometimes persist to eventually give a wide range of pathologies that includes a patent urachus, urachal cysts, sinuses, or diverticula.^4^ Residual tissues may also result in urachal adenocarcinoma, which is exceedingly rare, with an incidence from 0.01 to 0.2 per 100 000 population.^5^ Urachal adenocarcinoma constitutes around 0.5% to 2% of all bladder cancers.^6^ Due to its rarity and also due to the familiar clinical presentation overlapping with common bladder tumours, diagnosing it can be particularly challenging.^7^ Early and prompt diagnosis is indicated due to the aggressive nature of the disease in its advanced stage.^8^

## Case presentation

A 42-year-old male patient came with complains of haematuria, dysuria and burning micturition for 2 months. He is recently diagnosed with type II Diabetes 6 months and is on oral medications for the same. Urine analysis revealed haematuria and infection with 174 RBCs and 4 pus cells per high-power field. On clinical examination of the abdomen, there was no obvious palpable mass.

**Figure 1.**
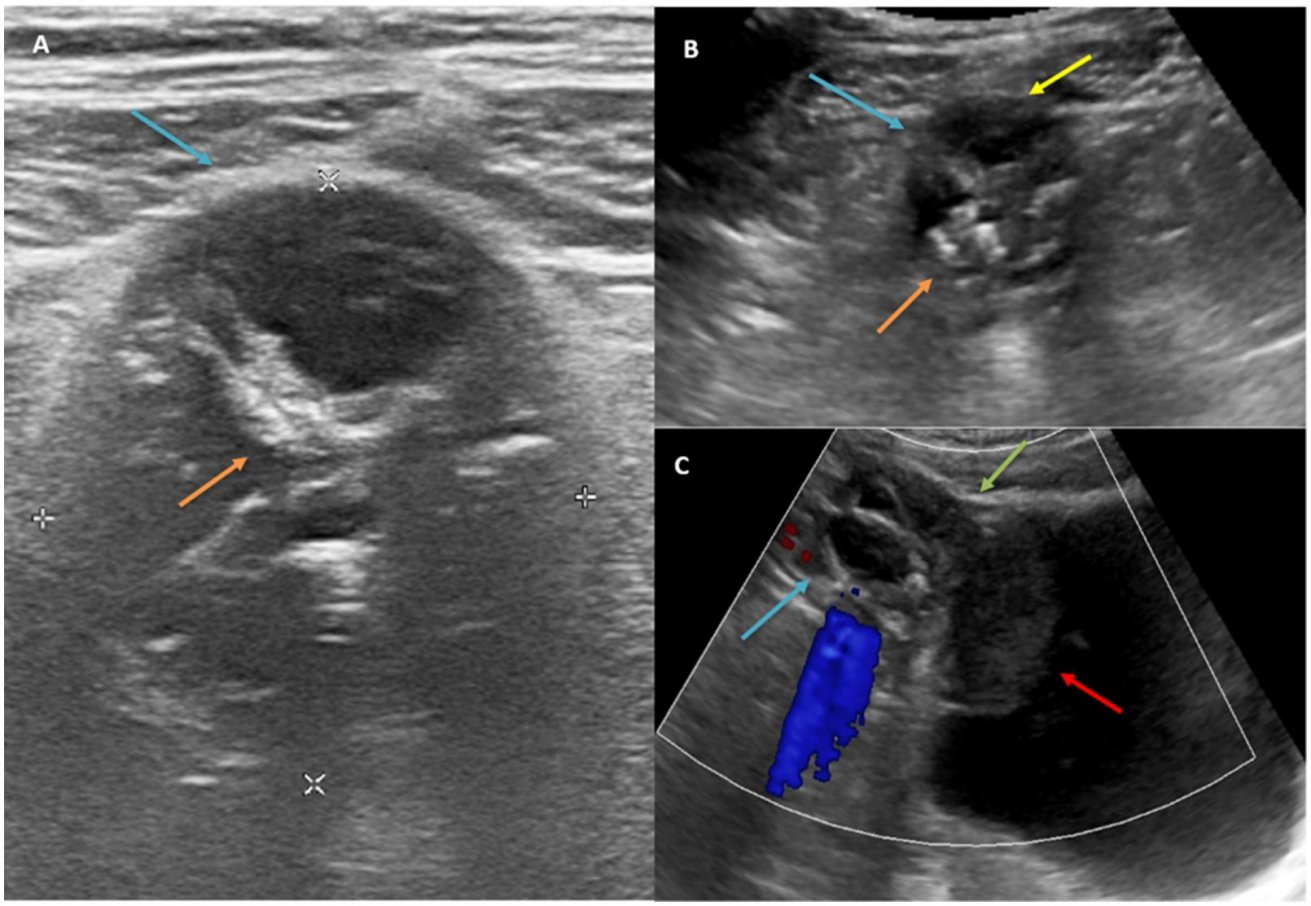
(A, B) axial and (C) sagittal USG shows a midline pelvic hypoechoic cystic lesion (blue arrows) with internal echoes and calcifications (orange arrow) located at the bladder dome (green arrow). The lesion extends cranially to the infraumbilical region near the anterior abdominal wall (yellow arrow) and inferiorly into the bladder as a polypoidal hyperechoic wall thickening (red arrow) with minimal vascularity and no positional variation.

On USG abdomen pelvis there is a well-defined round lesion with cystic areas, hyperechoic contents, calcifications throwing shadowing, and mild peripheral vascularity in the midline pelvis superior to the dome of the urinary bladder with infraumbilical extension cranially without involving the anterior abdominal wall and intravesical extension caudally in the form of polypoidal hyperechoic wall thickening/adherent contents without positional variation (Figure 1). Differentials diagnoses to consider at this stage were chronic infected urachal cyst with adherent clot/debris, Urachal Adenocarcinoma. To further characterise the lesion, CECT abdomen and chest was considered.

**Figure 2.**
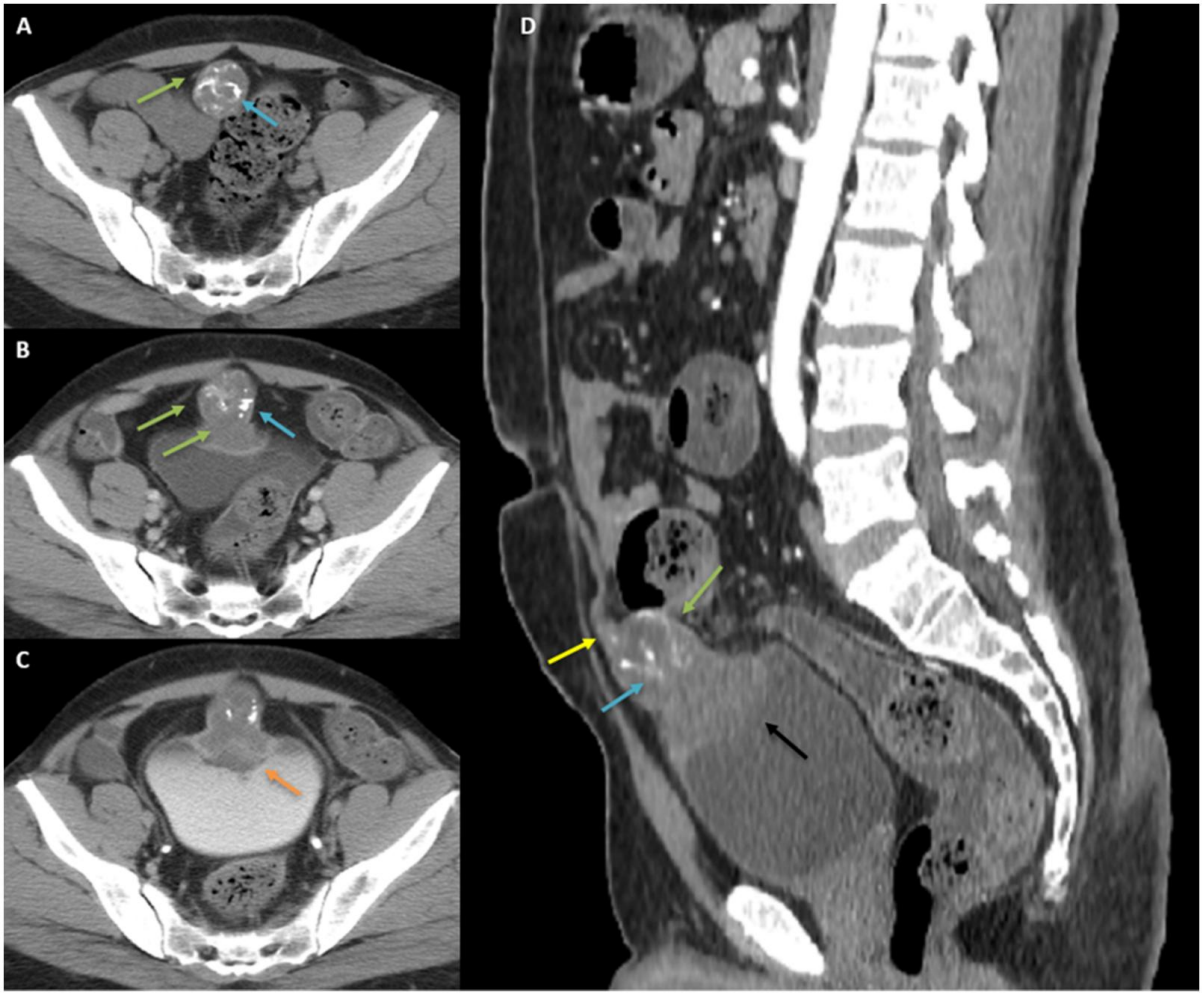
(A) Axial NCCT, (B) venous, (D) arterial phase CECT of abdomen and pelvis show a bell-shaped, heterogeneously enhancing infraumbilical mass (green arrow) with cystic/necrotic areas, hyperdense contents, septations, and calcifications (blue arrows). It is continuous with the urachal remnant (yellow arrow) antero-superiorly, without anterior abdominal wall involvement. Postero-inferiorly, it is infiltrating the bladder forming a polypoidal intravesical component (black arrow). CECT delayed phase (C) shows persistent enhancement of the lesion (orange arrow). No peritoneal, nodal, or distant metastases seen.

**Figure 3.**
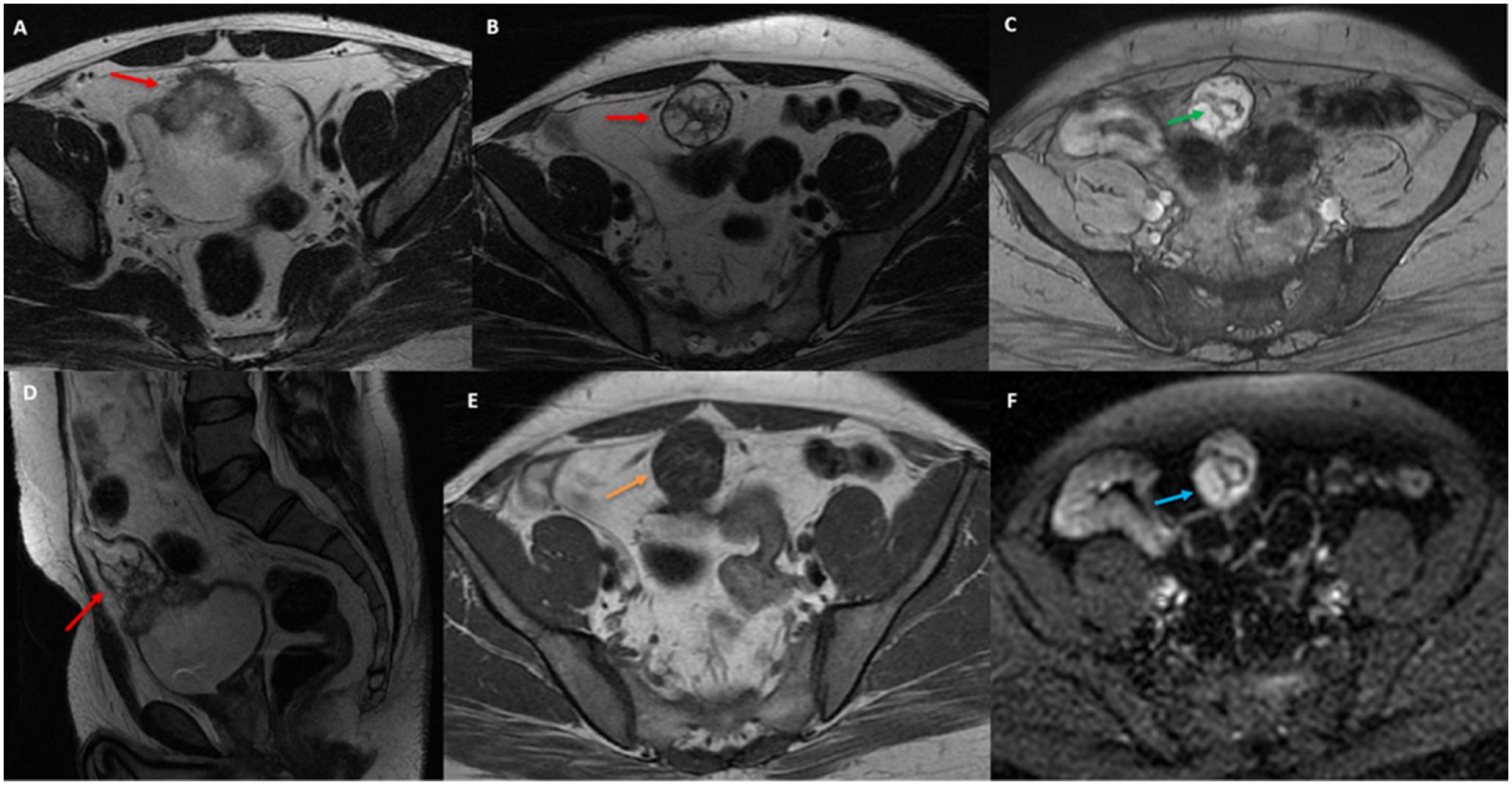
On MRI, (A, B) axial T2W, (D) Sagittal T2W sequences show a heterogeneously hyperintense mucinous lesion (red arrows) with hypointense wall, septations, and calcifications. (E) On axial T1W, the lesion appears heterogeneously hypointense (orange arrow). (C) GRE sequence shows blooming from calcifications (green arrow). DWI reveals restricted diffusion (blue arrow) indicating high cellularity, malignancy.

CECT of the abdomen and pelvis confirmed a well-defined bell-shaped heterogeneously enhancing soft tissue density lesion with few internal cystic/necrotic areas in the infraumbilical region with calcifications. Infraumblical extension along the urachal remnant and intravesical extension of the lesion were observed (Figure 2). No obvious involvement of the anterior abdominal wall, peritoneum, lymph node or distant metastasis was observed which was later verified on PET-CT. Persistent enhancement on delayed phase of CECT and heterogenous hyperintense signal on T2W and heterogenous hypointense signal on T1W images corresponds to its mucinous/fibrotic nature. Increased signal on DWI confirms its high cellularity/neoplastic nature. GRE blooming due to calcifications was also observed (Figure 3).

**Figure 4.**
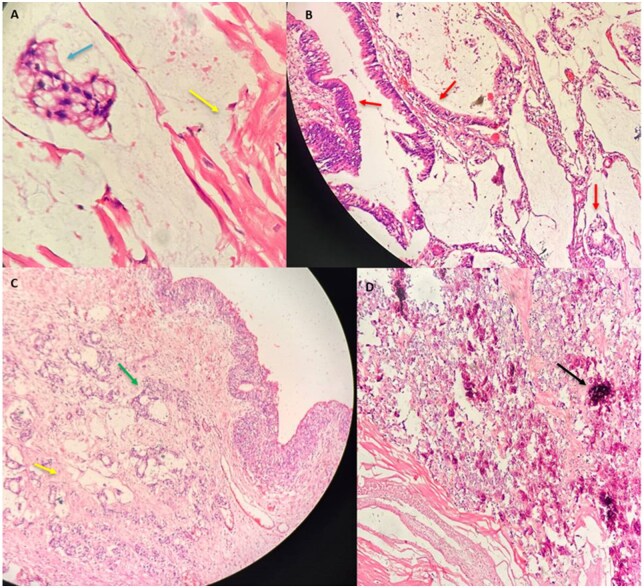
Histopathological examination with H & E (A) 100X (B-D) 40X reveals atypical malignant cells with hyperchromatic nuclei and eosinophilic cytoplasm in a glandular and villous pattern (red arrow) surrounded by extracellular mucin (yellow arrow). Signet ring cells (blue arrow) are also seen in the mucin pools. Calcifications (black arrow) and necrotic areas were seen. Tumour cells infiltrating the bladder wall, mucosa (green arrow) lined by transitional cell epithelium seen.

A final diagnosis of Urachal Adenocarcinoma invading the urinary bladder with no distant spread was made corresponding to Sheldon III A stage. Histopathology post resection confirmed its mucinous nature comprising of atypical malignant cells arranged in glandular pattern surrounding by large amounts of extracellular mucin. Signet ring cells were also noted in the mucin pool. Areas of calcification and necrosis were seen. Tumour cells in villous architecture were also seen involving the bladder wall and bladder mucosa lined by transitional cell epithelium (Figure 4). Patient underwent surgical resection of the urachus along with partial cystectomy followed by chemotherapy. On 6 month and yearly follow-ups, there was no recurrence of symptoms. There was no recurrence on follow up USG scans suggestive of good prognosis. However, patient could not complete chemotherapy course due to intolerance.

## Discussion

Symptoms usually include haematuria, observed in 60%-70% of cases, hence making it the most common symptom in cases of urachal adenocarcinoma.^9^ Other symptoms may be suprapubic pain, dysuria, and a palpable mass; however, these symptoms are less specific.^10^ Due to the nonspecific nature of these symptoms and the deep anatomical location of the tumour, they are generally diagnosed at an advanced stage, leading to a poor prognosis.^11^ Urachal adenocarcinoma can be associated with chronic inflammation, urachal remnants such as cysts or diverticula, and, in rare instances, with synchronous or metachronous malignancies of the gastrointestinal tract.^12^

Urachal adenocarcinomas are typically firm, irregular dome-based bladder masses with cystic degeneration, mucin, and calcifications. Histologically, they resemble gastrointestinal adenocarcinomas, complicating diagnosis. Tumour cells are arranged in glandular, tubular, or papillary patterns with marked atypia, mucinous cytoplasm, and occasionally signet ring cells.^3^ Subtypes include mucinous (most common), enteric, NOS, and signet ring cell types. Immunohistochemistry shows dual urothelial and gastrointestinal markers (CK7, CK20, CEA), aiding differential diagnosis. Prognosis depends on stage at presentation, with early-stage disease having better outcomes.^9^

Radiology plays a crucial role in the diagnosis, staging, and follow-up of urachal adenocarcinoma by aiding in the detection of the tumour’s location, extent, and potential metastasis.^10^ Imaging studies for these lesions include ultrasound, CECT, MRI and PET CT.^11^ Ultrasound, often the initial modality, can demonstrate a midline suprapubic mass with cystic, solid, and calcific components in the tumour.^5^ CECT is considered the gold standard, providing the most information about tumour size, extent, and involvement of surrounding structures.^12^ MRI, with its excellent soft-tissue contrast, allows for proper assessment of the tumour, its contents, and its relationship to surrounding structures.^13^ PET CT plays a key role in detecting metastatic disease, assessing tumour activity, residual/recurrent disease.

Urachal adenocarcinoma typically appears as a midline mass at the dome of the bladder on CT with mixed solid and cystic components.^14^ Calcifications, seen in approximately 50-70% of cases, are a cardinal diagnostic feature.^15^ MRI shows a hypointense mass on T1-weighted and hyperintense on T2-weighted images with diffusion restriction and variable enhancement after contrast administration.^6^ Imaging plays an essential role in staging, assessing local tumour invasion, lymph node involvement, and distant metastases, all of which are crucial for treatment planning.^7^

The Sheldon staging system is used to classify urachal adenocarcinoma as follows: Stage I—Tumour confined to urachal mucosa, Stage II—Tumour invades urachal musculature. In Stage III, the tumour extends beyond the urachus and is further subdivided into IIIA for invasion of the bladder, IIIB for invasion of the abdominal wall, and IIIC for invasion of the peritoneum.^1^ Stage IV involves lymph nodes or distant metastasis and is subdivided into IVA for regional lymph nodes and IVB for distant metastasis.^7^ The Mayo Clinic system further refines staging to incorporate surgical and pathologic findings, offering a more nuanced estimation of disease burden.^8^

Imaging differential diagnoses for urachal adenocarcinoma include primary bladder adenocarcinoma, metastatic adenocarcinoma from other sites such as the colon and ovaries, and benign urachal remnants with secondary infection.^9^ The involvement of the retropubic space of Retzius is frequently seen in urachal adenocarcinoma, differentiating it from primary bladder adenocarcinoma.^10^ Discriminating urachal adenocarcinoma from metastatic adenocarcinoma, particularly from the gastrointestinal tract, requires imaging alongside clinical and pathological correlation.^12^

Surgical resection is the first-line treatment for this aggressive cancer, typically involving partial or radical cystectomy with en bloc resection of the urachus and the umbilicus.^1^ Extended surgical resections are required when the tumour invades surrounding structures, potentially necessitating the resection of adjacent organs or tissues.^2^ Lymph node dissection is often performed to assess regional metastasis, which is crucial for staging and prognosis.^3^ Chemotherapy is commonly used in advanced cases, either as adjuvant therapy following surgery or for palliation in unresectable or metastatic disease and often involves agents such as 5-fluorouracil (5-FU) or platinum-based therapies.^4^

Urachal cancers are generally radioresistant, with radiotherapy used in only about 10% of cases. It may be considered for inoperable disease or positive margins, though evidence for benefit is limited.^13^Targeted therapies and newer immunotherapeutic agents are showing promise, particularly for tumours with high expression of distinct molecular markers, although their roles in urachal adenocarcinoma management are still under investigation.^7^ Poor prognostic factors include advanced-stage diagnosis, high-grade histology, and the presence of extensive local invasion or distant metastasis.^8^ Recurrence is common, especially in cases of incomplete tumour resection or late diagnosis, emphasising the importance of regular follow-up.^9^ Follow-up typically includes periodic imaging and, in some cases, cystoscopy, with more frequent monitoring in the first few years postoperatively when the risk of recurrence is highest.^10^

For patients with unresectable, recurrent, or very advanced disease, symptomatic management aims to improve quality of life.^11^ Because urachal adenocarcinoma is both complex and rare, a multidisciplinary approach involving urologists, oncologists, radiologists, and pathologists is necessary for optimal patient outcomes.^12^

## Conclusion

Radiologists play a pivotal role in the early recognition of characteristic imaging features and accurate diagnosis. Detailed imaging and pathology reports are crucial for effective staging and treatment planning. Given the rarity and aggressive nature of urachal adenocarcinoma, a multidisciplinary team comprising clinicians, urologists, radiologists, and pathologists is vital for managing these cases to optimise patient outcomes.^4^

## Data Availability

Data sharing not applicable- no new data generated.

## Learning points

- Urachal carcinoma is a rare bladder cancer, often presenting as a midline infraumbilical mass with peripheral calcifications.
- It mimics conditions like infected urachal remnants, diverticula, or transitional cell carcinoma.
- Radical cystectomy with en bloc resection is the treatment of choice.
- Late presentation due to extravesical origin leads to poor prognosis; early imaging-based diagnosis is crucial.
